# Association between social activities and risk of COVID-19 in a cohort of healthcare personnel

**DOI:** 10.1017/ash.2024.485

**Published:** 2025-01-30

**Authors:** Holly Shoemaker, Haojia Li, Yue Zhang, Jeanmarie Mayer, Michael Rubin, Candace Haroldsen, Morgan M. Millar, Per H. Gesteland, Andrew T. Pavia, Lindsay T. Keegan, Jessica Marie Cole, Egenia Dorsan, Matthew Doane, Kristina Stratford, Matthew Samore

**Affiliations:** 1 Department of Population Health Sciences, Spencer Fox Eccles School of Medicine, University of Utah, Salt Lake City, UT, USA; 2 IDEAS Center of Innovation, Veterans Affairs Salt Lake City Health Care System, Salt Lake City, UT, USA; 3 Division of Epidemiology, Spencer Fox Eccles School of Medicine, University of Utah, Salt Lake City, UT, USA; 4 Department of Veterans Affairs, VA Salt Lake City Healthcare System, Salt Lake City, UT, USA; 5 Division of Pediatric Hospital Medicine, Spencer Fox Eccles School of Medicine, University of Utah, Salt Lake City, UT, USA; 6 Utah Education Policy Center, University of Utah, Salt Lake City, UT, USA

## Abstract

**Objective:**

Previous studies have linked social behaviors to COVID-19 risk in the general population. The impact of these behaviors among healthcare personnel, who face higher workplace exposure risks and possess greater prevention awareness, remains less explored.

**Design:**

We conducted a Prospective cohort study from December 2021 to May 2022, using monthly surveys. Exposures included (1) a composite of nine common social activities in the past month and (2) similarity of social behavior compared to pre-pandemic. Outcomes included self-reported SARS-CoV-2 infection (primary)and testing for SARS-CoV-2 (secondary). Mixed-effect logistic regression assessed the association between social behavior and outcomes, adjusting for baseline and time-dependent covariates. To account for missed surveys, we employed inverse probability-of-censoring weighting with a propensity score approach.

**Setting:**

An academic healthcare system.

**Participants:**

Healthcare personnel.

**Results:**

Of 1,302 healthcare personnel who completed ≥2 surveys, 244 reported ≥1 positive test during the study, resulting in a cumulative incidence of 19%. More social activities in the past month and social behavior similar to pre-pandemic levels were associated with increased likelihood of SARS-CoV-2 infection (recent social activity composite: OR = 1.11, 95% CI 1.02–1.21; pre-pandemic social similarity: OR = 1.14, 95% CI 1.07–1.21). Neither was significantly associated with testing for SARS-CoV-2.

**Conclusions:**

Healthcare personnel social behavior outside work was associated with a higher risk for COVID-19. To protect the hospital workforce, risk mitigation strategies for healthcare personnel should focus on both the community and workplace.

## Introduction

Healthcare personnel (HCP) are essential workers on the front lines during public health emergencies. Infections in HCP during epidemics, whether from workplace or community exposure, can place strain on healthcare systems and adversely impact patient care through inadequate staffing, causing higher caseloads, longer work hours, potential burnout, and generally increased workplace strain.^1–8^ It is therefore critically important to minimize infections in HCP, especially during disease surges.

While it was shown that HCP experienced a higher risk of SARS-CoV-2 infection from workplace exposures early in the COVID-19 pandemic,^9–11^ over time, infection among HCP has become primarily driven by community exposures.^12–16^ Several studies on community risk found specific social activities or venues to be associated with SARS-CoV-2 exposure,^17–19^ but those studies focused on general community risk and may not be widely generalizable to the risk posed to HCP, due to HCP increased awareness of risk,^20^ or pandemic-related emotional exhaustion.^21^ To date, sufficient research has not been conducted to thoroughly examine the association between HCP social activity and risk of infection. This information is needed in order to adequately plan precautionary measures in the event of future disease outbreaks.

To better understand the behaviors of HCP in the community and the association of these behaviors with risk of SARS-CoV-2 infection, we conducted a longitudinal study over 6 months during the anticipated viral respiratory season, which corresponded with the Omicron surge. This study presents a unique opportunity to examine HCP behaviors in the community and the associated COVID-19 risk.

## Methods

### Setting & participants

We recruited HCP working in patient care areas in an academic healthcare system. Recruitment occurred through email, flyers, and clinical livestreams. Participation in the survey was voluntary and without compensation. Informed consent was obtained from all participants prior to the administration of the survey. The study was approved by the institutional review board of the University of Utah (IRB_00145823).

### Survey

We conducted a prospective cohort study from December 2021 to May 2022. There were in total three types of surveys: the baseline survey, monthly surveys, and the final survey. When the participants initially completed the survey, they completed a one-time baseline survey and a monthly survey. The baseline survey collects primarily demographic information, HCP work role, and location. The monthly survey included questions about viral symptoms, vaccination, prior self-reported testing for SARS-CoV-2 and results, and attitudes and behaviors related to COVID-19. Thirty days after a participant submitted a survey, an email or text was sent to request the participant to complete a follow-up monthly survey with the same questions as asked in the initial one. In late May, we sent out the final survey, as well as another monthly survey. We excluded this round of monthly surveys from our analysis because a large proportion of participants completed the monthly survey twice within 30 days, resulting in duplicated responses.

### Exposures

We used two primary self-reported exposures for social activities. The first exposure, *recent social activity composite*, was a composite of social activities in the past month as reported in each survey. The survey queried nine individual social activities, asking whether the participant in the last 30 days: (1) socialized at home indoors with non-household members; (2) socialized outdoors with non-household members; (3) socialized or attended an indoor event in public (i.e., concert, movies, sporting); (4) socialized or attended an outdoor event in public (i.e., concert, movies, sporting); (5) attended in-person religious services; (6) went to a store (i.e., grocery, retail, etc.); (7) went to a gym or fitness center; (8) ate indoors at a restaurant; (9) traveled on an airplane. We combined individual activities into the combined score, recent social activity composite, on a scale of 0 to 9. We utilized logistic principal component analysis to justify the appropriateness of combining social activities into one score.

The second self-reported exposure, *pre-pandemic social similarity,* compared current social activity to pre-pandemic social activity. The original question in the survey asked “Compared to your typical pre-pandemic social activities (prior to March 2020), how limited are your current social activities? Select a number that best corresponds on a scale from 0 (not at all limited, activities comparable to pre-pandemic) to 10 (extremely limited).” To ensure parallel direction with the other exposure and facilitate clearer interpretation of the effects, we reversed participants’ limitation ratings and converted them into a measure of social similarity. The pre-pandemic social similarity ranged from 0 (activities were extremely limited), to 10, (social engagement was completely comparable to pre-pandemic levels).

### Outcomes

The primary outcome was a self-reported positive test for SARS-CoV-2 since the previous survey. The secondary outcome was undergoing SARS-CoV-2 testing since the previous survey, regardless of a positive or negative test result.

### Covariates

The survey collected both baseline and time-dependent covariates, which might be potential confounders. Baseline covariates included age, gender, clinical role, work location, self-rated health, comorbidities, and household condition. Time-dependent covariates included the calendar month of taking the survey, time since the prior survey, time since the most recent COVID-19 vaccine, time since the most recent SARS-CoV-2 infection, and illness reported since the preceding survey. We treated all the covariates, except for the time since the prior survey, as potential effect modifiers.

We collapsed the categories of clinical roles based on similar clinical responsibilities and patterns of social activity. The clinical roles included: (1) nurse; (2) nurse assistant, including certified nurse assistant, healthcare assistant, medical assistant, or emergency medical technician; (3) physician and advanced practice clinician such as physician assistant; and (4) other, including physical therapist, respiratory therapist, pharmacist, patient relations specialist, technologist, and the “Other (please specify)” category.

The question regarding work location asked the typical location which the participant most often worked in. We grouped the responses into the following categories: (1) outpatient ambulatory, including ambulatory clinic and urgent care; (2) inpatient acute care, including acute care and inpatient rehab unit within the academic healthcare system; (3) critical care, including critical care units and emergency department, (4) inpatient psychiatry, and (5) other, including perioperative or operating room (OR), and “Other (please specify).”

We combined comorbidities as one binary indicator of whether the HCP had any diabetes, hypertension or high blood pressure, cardiovascular disease or heart disease, pulmonary disease, any condition requiring immunosuppressive therapy, immunocompromised condition, autoimmune disease, and/or kidney disease. The household conditions included three categories: (1) live alone, (2) live with others, no school-age children (under age 12), and (3) live with others, at least one school-age child.

### Statistical analysis

We summarized the distribution of HCP characteristics at the baseline survey using count and percentage. For responses of “Prefer not to answer” for age, gender, and self-reported health, and transgender and non-binary responses to gender, we treated them as missing values, and excluded them from the regression analysis, due to the small sample sizes.

We estimated the cumulative incidence of SARS-CoV-2 infection using the Kaplan-Meier failure function. We implemented a 90-day washout period, setting the index date for each participant as either December 1^st^, 2021, or 90 days after a SARS-CoV-2 infection reported between September 2^nd^ and November 30^th^, 2021. If no SARS-CoV-2 infection date was reported during the study, the censoring date was designated as the end of the study period, May 31^st^, 2022. Given the rarity of participants experiencing multiple infections within the study timeframe, the calculation of the cumulative incidence rate only considered the first SARS-CoV-2 infection reported by each participant. To assess the impact of the Omicron variant on social activity, we calculated the monthly percentages for each individual recent social activity and illustrated their temporal trends. We employed unadjusted logistic mixed effects regression models to determine whether changes from January to May, relative to December.

We employed mixed-effect logistic regression to estimate the associations of self-reported exposure measures with the outcomes after adjusting for the confounding covariates. In the mixed-effect models, we accounted for the clustering effect among the repeatedly measured survey by using an HCP-level random effect. We used the inverse probability-of-censoring weight with the covariate balancing propensity score approach to calculate the propensity of missing the scheduled survey and account for missed surveys and loss-to-follow-up during the study. This compensated for censored participants by giving more weight to observations with similar characteristics to those not censored. We calculated standardized mean differences to check the covariate balance after the adjustment of inverse probability-of-censoring weight.

In both the outcome regression and covariate balancing propensity score model, we incorporated time-independent and time-dependent covariates. To ensure that time-dependent predictors, including the exposures and the covariates, preceded the outcome and censored events in time, thereby preserving the correct temporal order for precise analysis, we used responses from the previous survey to predict outcomes in the current survey. To test whether the relationships between exposures and outcomes were linear, we fitted cubic polynomial models.

As a secondary analysis, we also used the same regression method to evaluate the association of each individual social activity with testing positive for SARS-CoV-2 and estimated the group-specific effect of social behavior by covariates. The likelihood ratio tests were performed to evaluate the presence of effect modification.

Statistical analyses were conducted using R (version 4.4.0), with statistical significance level of alpha = .05.

## Results

Recruitment emails were sent to 10,321 HCP; 1,802 (18% of 10,321) consented to participate. More than two-thirds (72% 1,302/1,802) of HCP completed two or more monthly surveys, with an average of three surveys per participant. Participants were predominantly female (79%) and aged between 25 to 54 years (78%) (Table 1). Nurses represented the largest clinic role group at 33%, followed by “Other” at 29%. A plurality worked in outpatient ambulatory settings (37%). Most HCP rated their health condition as “excellent” or “very good” (73%), and 23% reported at least one comorbidity listed in the questionnaire. Additionally, 30% of HCP reported living with at least one school-aged child.

**Table 1.** Summary of healthcare personnel characteristics at baseline survey

Characteristic	N = 1,302^1^
Age in years	
18–24	98 (7.5%)
25–34	368 (28%)
35–44	386 (30%)
45–54	255 (20%)
55–64	155 (12%)
65+	39 (3.0%)
Missing	1
Gender	
Male	270 (21%)
Female	1,020 (79%)
Missing	12
Clinic role	
Nurse	434 (33%)
Nurse assistants (CNA, HCA, MA, or EMT)	215 (17%)
Physician/APC	273 (21%)
Other	380 (29%)
Work location	
Outpatient ambulatory	481 (37%)
Inpatient acute care	206 (16%)
Critical care	209 (16%)
Inpatient psychiatry	39 (3.0%)
Others	367 (28%)
Self-rated health	
Excellent	340 (26%)
Very good	616 (47%)
Good or below	342 (26%)
Missing	4
Any comorbidities	301 (23%)
Household condition	
Living alone	142 (11%)
Living with others, no school-age children	767 (59%)
Living with others, 1+ school-age children	393 (30%)

1 n (%).“Missing” consisted of responses of “Prefer not to answer” for age, gender, and self-reported health, and transgender and non-binary responses to gender.

Comorbidities include diabetes, hypertension or high blood pressure, cardiovascular disease or heart disease, pulmonary disease, any condition requiring immunosuppressive therapy, immunocompromised condition, autoimmune disease, and kidney disease.

**Figure 1.**
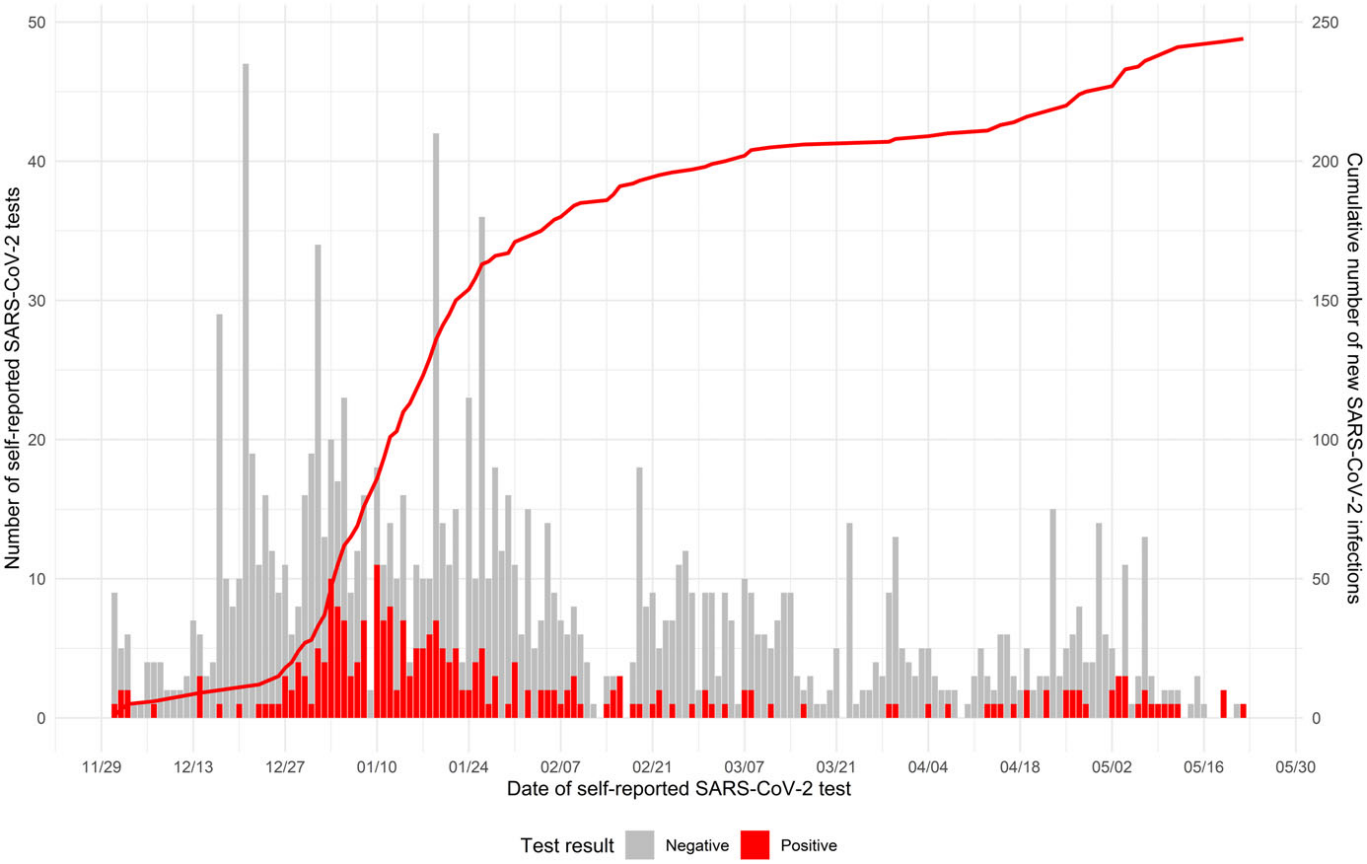
Self-reported SARS-CoV-2 test results over time. The left y-axis corresponds to the number of self-reported SARS-CoV-2 test results, represented by the gray bars for negative results and the red bars for positive results. The right y-axis tracks the cumulative number of new SARS-CoV-2 infections, depicted by the red line curve.

As of December 1^st^, 2021, 210 (16%) of 1,302 HCP reported already having had COVID-19 at their baseline survey. Throughout the study period from December 1^st^, 2021, to May 31^st^, 2022, 244 HCP reported a SARS-CoV-2 infection since the last survey, leading to a cumulative incidence of 19% (Fig. 1). Two HCP reported a second SARS-CoV-2 infection, which was not included in the calculation of the cumulative incidence. The peak of new infections in participating HCP occurred during January and February, corresponding to the peak in SARS-CoV-2 infections in Utah.

**Figure 2.**
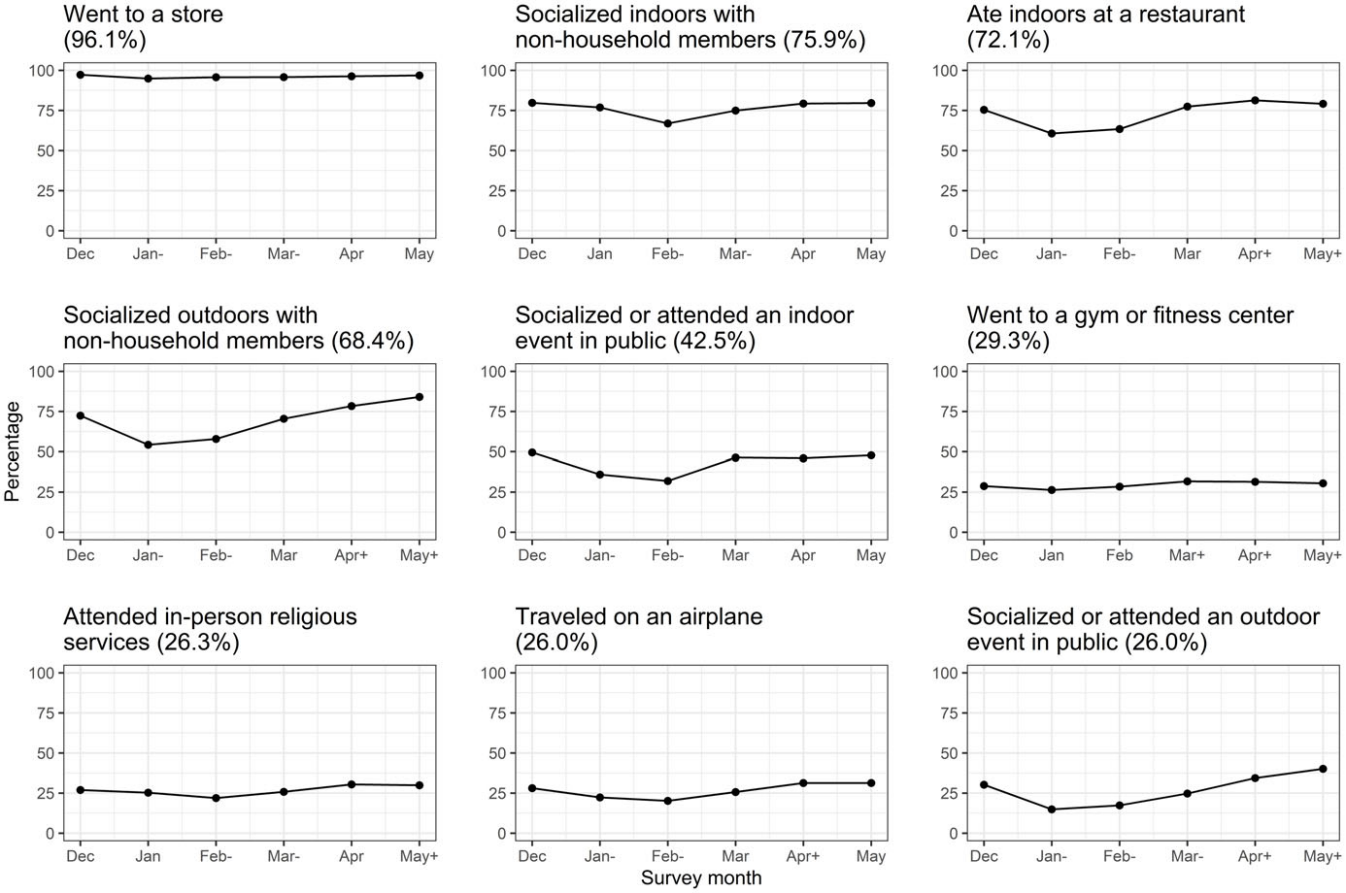
Temporal trends of individual social activities. Social activities were ordered by the overall rate. The months on the x-axis refer to the time when the participants completed the survey, reflecting the social activities they had engaged in during the preceding months. Statistically significant changes in the prevalence of social activity compared to the baseline (December) are indicated by “+” for increases and “−” for decreases, appended to the survey month.

The percentage of reported social activities varied throughout the survey time period (Fig. 2). All the individual social activities in the preceding month, except for going to a gym or fitness center, as reported in January and/or February, significantly decreased, reflecting the impact of the Omicron surge from December 2021 to January 2022. Physicians reported a greater decrease in eating at restaurants during the peak of Omicron activity but changes in behaviors were generally similar by role (Figure S2).

As the first component of logistic principal component analysis explained a large proportion of variation and the factor loading of each individual social activity was comparable with the same direction (Table S2), we determined that a combined score was reasonable. The distribution of the two self-reported exposure measures, recent social activity composite and pre-pandemic social similarity showed a great deal of variation (Figure S1). There was a moderate positive correlation between the two measures of exposures, with a Pearson’s correlation coefficient of .47.

**Figure 3.**
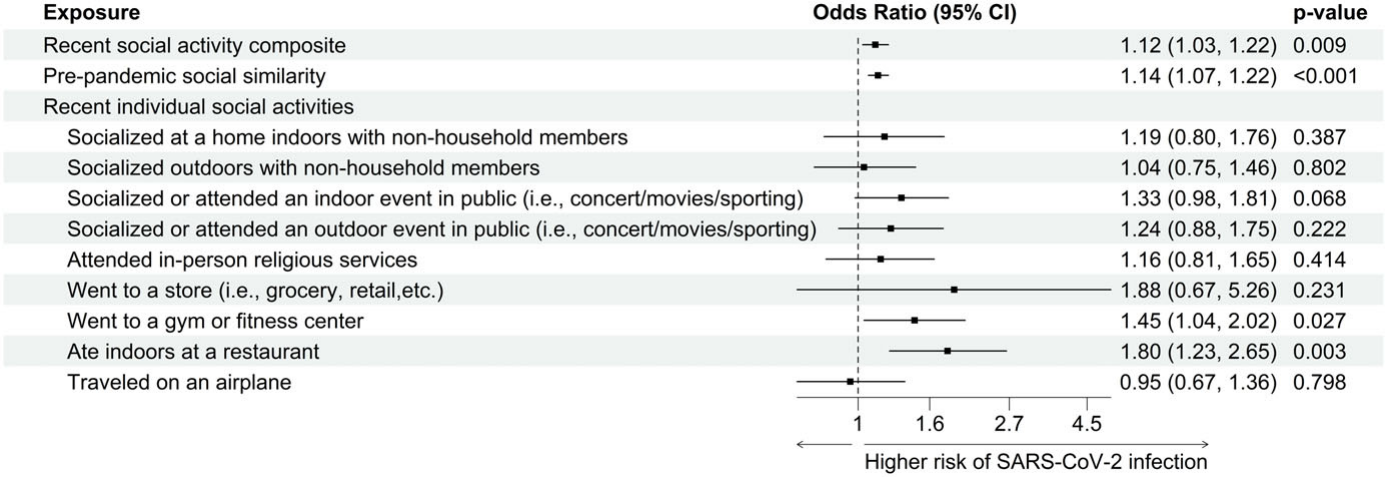
Effect of recent social activity composite, pre-pandemic social similarity, and recent individual social activities, on SARS-CoV-2 infection among healthcare personnel. Covariates included in the models: age, gender, clinical role, work location, self-rated health, any comorbidities, household condition, calendar month of taking survey, months since last survey, months since recent COVID vaccine, months since recent SARS-CoV-2 infection, and recent illness.

All covariates were balanced after being adjusted by inverse probability-of-censoring weight (Figure S3). Higher recent social activity composite and pre-pandemic social similarity reported in the previous survey were significantly associated with an increased likelihood of positive test result for SARS-CoV-2 in the adjusted model (recent social activity composite: OR = 1.12, 95% CI 1.03–1.22, *P* = .009; pre-pandemic social similarity: OR = 1.14, 95% CI 1.07–1.22, *P* < .001) (Fig. 3). The details of the full regression model, including coefficients of exposures and covariates, are summarized in Table S1. None of the second- or third-order terms in the cubic polynomial models were significant, indicating that the relationships between exposures and outcomes were linear. Neither of the exposure measures was significantly associated with undergoing testing for SARS-CoV-2 (recent social activity composite: OR = 1.01, 95% CI .96–1.07, *P* = .661; pre-pandemic social similarity: OR = .98, 95% CI .94–1.02, *P* = .332; results not shown in tables or figures). Going to a gym or fitness center and dining indoors at a restaurant were each significantly associated with a higher risk of COVID-19 (OR = 1.45, 95% CI 1.04–2.02, *P* = .027, and OR = 1.80, 95% CI 1.23–2.65, *P* = .003, respectively). Attending a concert or sporting event was associated with a trend of a higher risk of infection but the confidence interval crossed one.

The group-specific effects of the recent social activity composite and pre-pandemic social similarity on SARS-CoV-2 infection are summarized as forest plots in Figures S4 and S5, respectively, where each point represents an estimated effect size in a subgroup, with horizontal lines indicating the 95% confidence intervals. Based on the interaction *P*-values from the likelihood ratio test, none of the covariates were found to be significant effect modifiers.

## Discussion

Our longitudinal survey study of HCP found that risk of COVID-19 was associated with two different global measures of social behavior. One measure was a novel index of similarity of current social activities to pre-pandemic social activities and the other was a sum of individual social behaviors. Additionally, we found that specific individual activities, including eating indoors at a restaurant and going to a fitness center, were associated with increased risk of positive SARS-CoV-2 test. The likely explanation for these findings is that HCP who engaged in more social activities experienced increased exposure to infectious individuals. Taken together, these results suggest that collection of self-reported data on infection and behavior can be used to identify epidemiologically meaningful exposures.

Our findings are supported by previous studies that examined SARS-CoV-2 infection risk from composite social activities. For example, Arashiro et al.^17^ found that those who reported more social activities in the two weeks preceding a SARS-CoV-2 test were more likely to test positive among outpatients in Japan. However, we are unaware of any studies that examine COVID-19 risk with regard to the similarity of behavior to pre-pandemic levels. Our finding that eating indoors at a restaurant carried the strongest association with risk of COVID-19 is consistent with previously reported studies. Multiple studies have found eating in restaurants^17,19,22–24^ or going to a fitness center^17,18^ to be a risk factor for SARS-CoV-2 infection among the general population, although some have reported conflicting results with dining at restaurants.^18^ Indoor restaurants may involve large gatherings of people in tight spaces, and the consumption of food makes masking difficult. Moderate-intensity exercise, as might be seen at a fitness center, is associated with substantially increased aerosol particle production^25^ and could result in increased risk of transmission.

We found evidence that HCP decreased their social activities during the peak of the Omicron surge, suggesting that behaviors were modulated by perceptions of risk. HCPs were expected to use appropriate personal protective equipment and adhere to other recommended preventative measures for respiratory viral illness in healthcare settings during the pandemic,^26^ but other groups have reported that compliance with preventative measures varied wildly.^27^ HCPs may take different precautions inside and outside of work, and may also have different risk tolerance while at work or when in the community setting. Of interest, in our study, social behaviors were not associated with obtaining a test for SARS-CoV-2.

While HCPs face the risk of infection while performing their occupational duties, previous research has found that HCPs are more likely to have sources of SARS-CoV-2 infection in the community than in their workplace.^12^ Our study supports recommendations for HCP to reduce their risk of non-occupational infection. An option previously suggested within hospitals includes strategically implementing preventative measures as rates of disease rise or lower in the community, or during specific high-risk times of the year.^28^ Methods to reduce community risk in HCP during outbreaks could include staying up to date on vaccination,^29^ encouraging masking^24,30^ in the community during outbreaks, and ensuring accessible and affordable testing^31^ for all HCP.

This study has several limitations. Notably, our data are all self-reported and may be subject to recall bias or social desirability bias. Our study population may not reflect all who were contacted, as only a portion of those sent recruitment emails responded and consented to participate. While we know how many activities are reported, we do not know their duration. However, we anticipate that individuals with a greater number of reported activities likely spent more time at them compared to those with fewer. We have also done our best to estimate the infection date based on reported symptoms and the timing of previous surveys. Some individuals may have been infected but never tested, but we were only capable of analyzing those who were tested. Another limitation is that we did not ask about household contacts with COVID-19, but we have assessed for effect modification of household size under the idea that single individual households will not have household contacts and found no significant effect modification. Finally, participants may have engaged in preventative behaviors while engaging in social activities in the community, such as masking, which have been shown to reduce the risk of SARS-CoV-2 infection.^30^ We did not ask about these behaviors, but the impact of preventative behaviors in HCP while engaging in social activities could be a focus of future studies.

This study, which overlapped with the Omicron surge, identified HCP participation in a range of social activities. Reporting levels of social activity similar to pre-pandemic levels and a higher number of activities were associated with an increased risk of subsequent COVID-19. As cases of COVID-19 become increasingly underreported through the use of home test kits, longitudinal studies of infection risk like the current study become more important, and should continue to be pursued in the future. To protect the hospital workforce, especially when respiratory virus prevalence is high, HCP’s risk mitigation strategies should focus on the community and workplace. Future outbreaks of respiratory illness are inevitable; it is essential that we prepare through additional research and planning of interventions.

## Supporting information

Shoemaker et al. supplementary material Shoemaker et al. supplementary material

## Supplementary material

For supplementary material accompanying this paper visit http://doi.org/10.1017/ash.2024.485. click here to view supplementary material
