# Supplementary material for: Association between social activities and risk of COVID-19 in a cohort of healthcare personnel
Source: Antimicrob Steward Healthc Epidemiol. 2025 Jan 30;5(1):e29. doi: 10.1017/ash.2024.485 (PMC11795425; doi:10.1017/ash.2024.485)

**Supplemental**

**Title**: Association Between Social Activities and Risk of COVID-19 in a Cohort of Healthcare Personnel

Table of Contents

[Figures 2](#_Toc170334856)

[Figure S1. Overall distribution of social activity composite and pre-pandemic social similarity. 2](#_Toc170334857)

[Figure S2. Temporal trends of individual social activities among roles. 3](#_Toc170334858)

[Figure S3. Covariate balance 4](#_Toc170334859)

[Figure S4. Group-specific effects of recent social activity composite on SARS-CoV-2 infection 5](#_Toc170334860)

[Figure S5. Group-specific effects of pre-pandemic social similarity on SARS-CoV-2 infection 6](#_Toc170334861)

[Tables 7](#_Toc170334862)

[Table S1.  Estimated coefficients table of full regression models on SARS-CoV-2 infection 7](#_Toc170334863)

[Table S2. Principal component analysis (PCA) loadings for social activities 9](#_Toc170334864)

[Survey Instrument 10](#_Toc170334865)

[Baseline Survey 10](#_Toc170334866)

[Monthly Survey 16](#_Toc170334867)

# Figures

## Figure S1. Overall distribution of social activity composite and pre-pandemic social similarity.


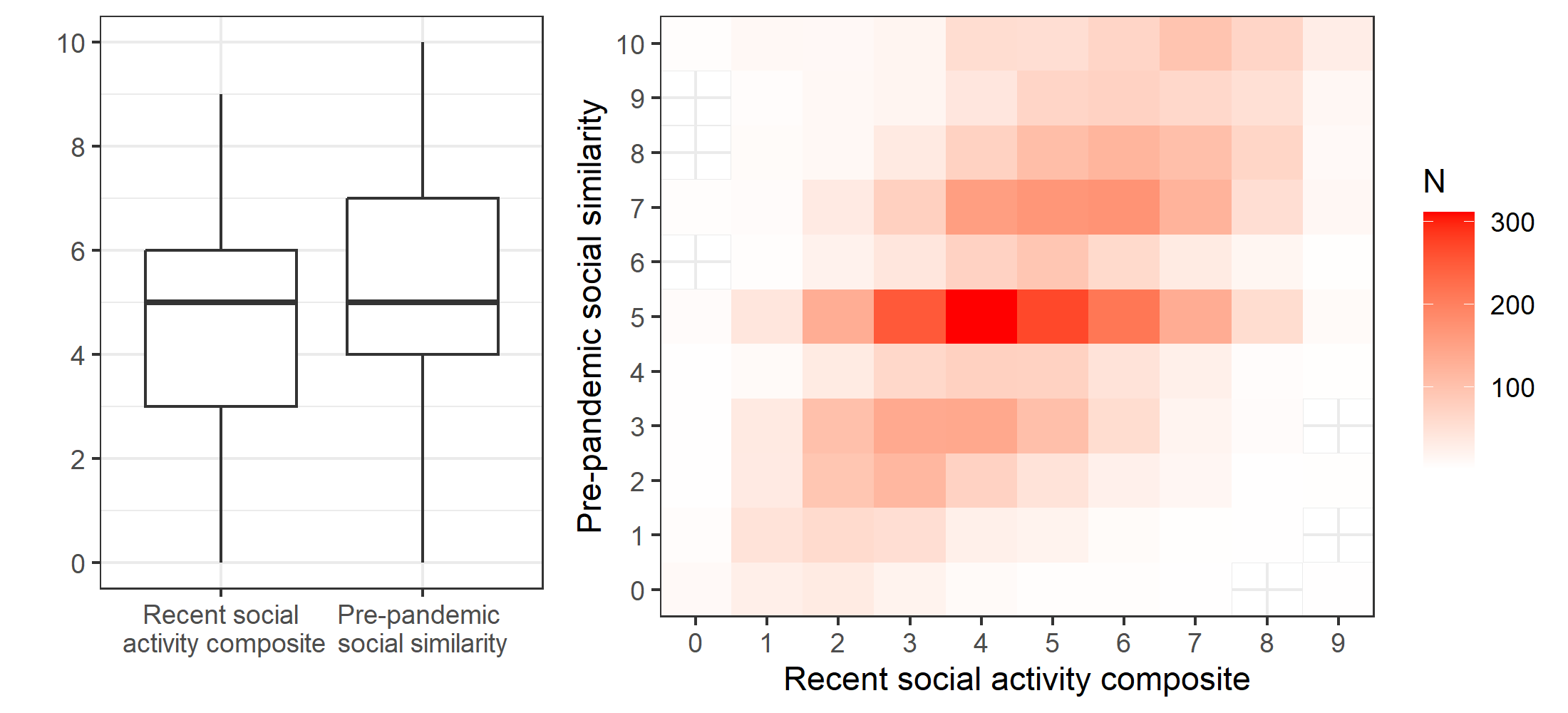


Figure S1. Overall distribution of social activity composite and pre-pandemic social similarity. There was a moderate positive correlation between the two exposures, with Pearson’s r = 0.47.

## Figure S2. Temporal trends of individual social activities among roles.


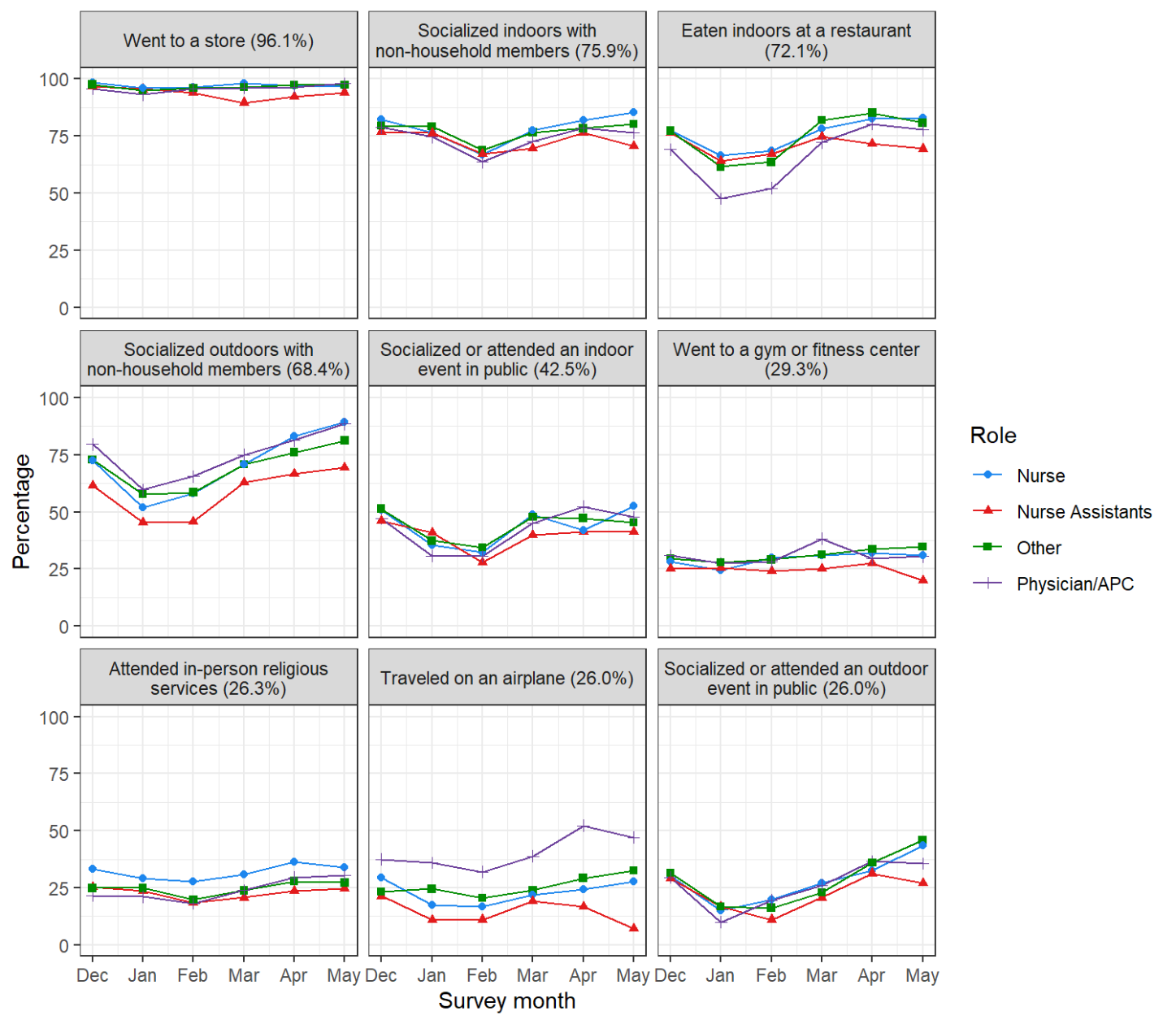


Figure S2. Temporal trends of individual social activities among roles. Social activities were ordered by the overall rate.

## Figure S3. Covariate balance


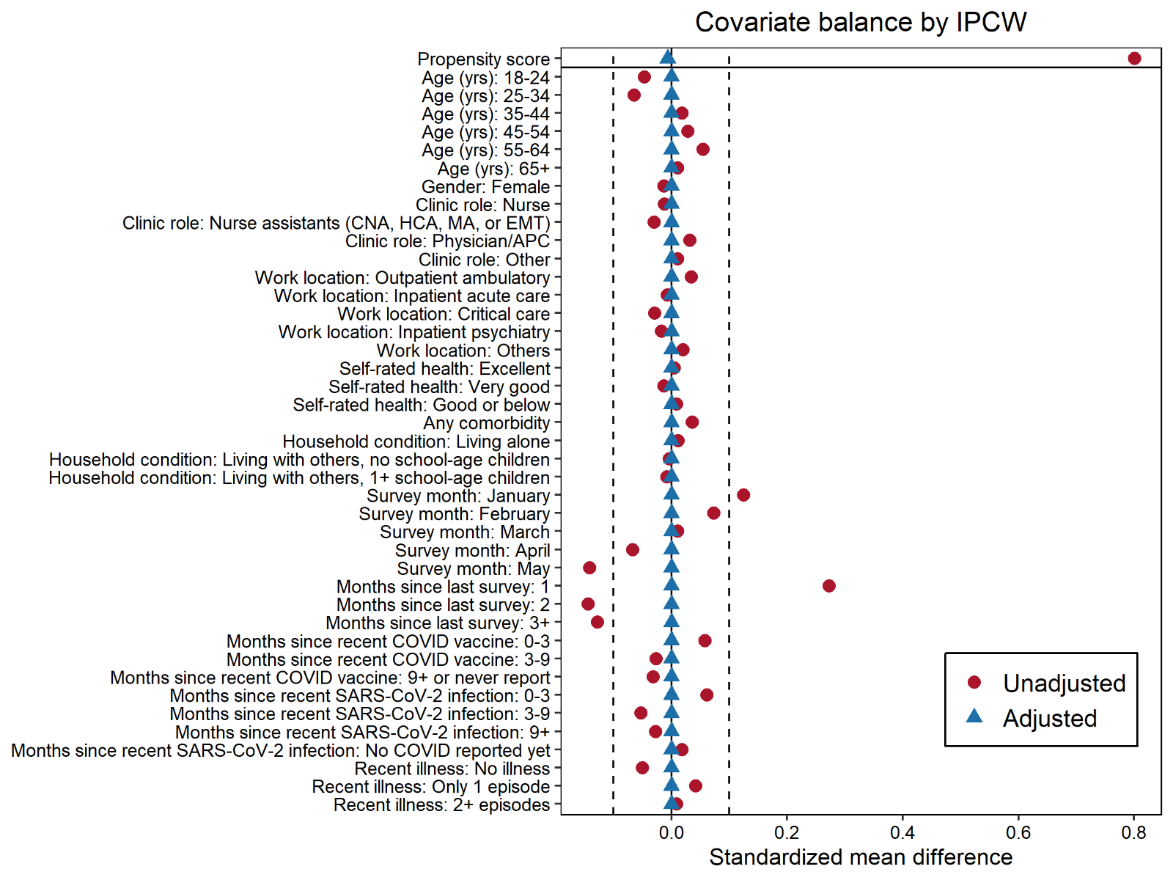


Figure S3. Covariate balance is adjusted by the inverse probability of censoring weight using the covariate balancing propensity score method. The dashed lines at -0.1 and 0.1 serve as the threshold, indicating an acceptable level of covariate balance.

## Figure S4. Group-specific effects of recent social activity composite on SARS-CoV-2 infection


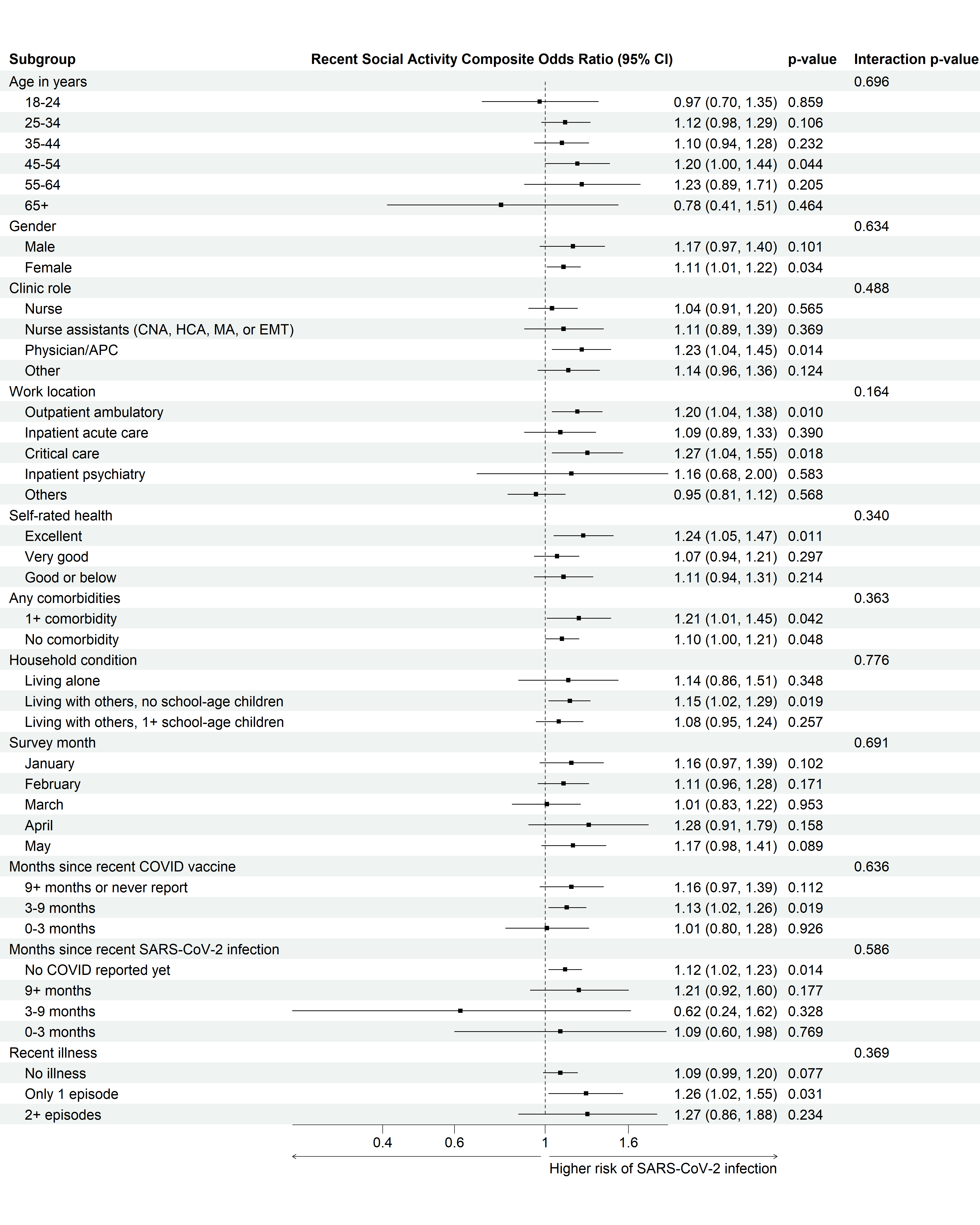


## Figure S5. Group-specific effects of pre-pandemic social similarity on SARS-CoV-2 infection


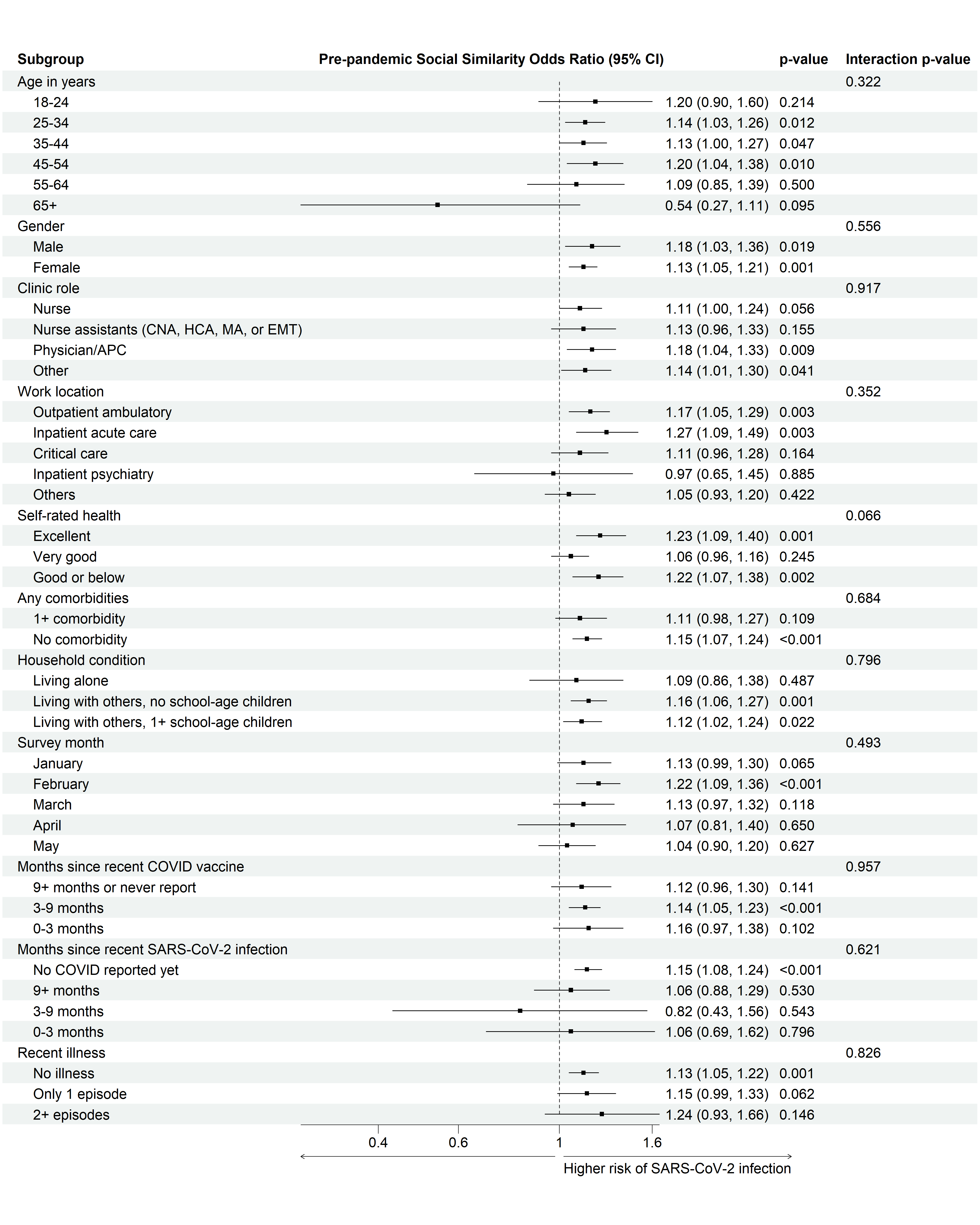


# Tables

## Table S1.  Estimated coefficients table of full regression models on SARS-CoV-2 infection

|  | Exposure | | | | | |
| --- | --- | --- | --- | --- | --- | --- |
|  | Recent social activity composite | | | Pre-pandemic social similarity | | |
| Characteristics | OR | 95% CI | p-value | OR | 95% CI | p-value |
| Recent social activity composite | 1.12 | 1.03, 1.22 | 0.009 | — | — |  |
| Pre-pandemic social similarity | — | — |  | 1.14 | 1.07, 1.22 | <0.001 |
| Age in years |  |  |  |  |  |  |
| 18-24 | — | — |  | — | — |  |
| 25-34 | 2.21 | 1.08, 4.53 | 0.030 | 2.13 | 1.04, 4.38 | 0.039 |
| 35-44 | 1.27 | 0.58, 2.77 | 0.6 | 1.21 | 0.56, 2.65 | 0.6 |
| 45-54 | 1.24 | 0.56, 2.74 | 0.6 | 1.20 | 0.54, 2.64 | 0.7 |
| 55-64 | 0.72 | 0.28, 1.89 | 0.5 | 0.66 | 0.25, 1.71 | 0.4 |
| 65+ | 0.71 | 0.18, 2.83 | 0.6 | 0.65 | 0.16, 2.57 | 0.5 |
| Gender |  |  |  |  |  |  |
| Male | — | — |  | — | — |  |
| Female | 0.97 | 0.66, 1.42 | 0.9 | 0.96 | 0.66, 1.41 | 0.8 |
|  |  |  |  |  |  |  |
| Nurse | — | — |  | — | — |  |
| Nurse assistants (CNA, HCA, MA, or EMT) | 0.83 | 0.49, 1.40 | 0.5 | 0.77 | 0.46, 1.30 | 0.3 |
| Physician/APC | 1.49 | 0.97, 2.31 | 0.070 | 1.52 | 0.99, 2.35 | 0.058 |
| Other | 0.79 | 0.53, 1.19 | 0.3 | 0.80 | 0.53, 1.21 | 0.3 |
| Work location |  |  |  |  |  |  |
| Outpatient ambulatory | — | — |  | — | — |  |
| Inpatient acute care | 1.16 | 0.74, 1.81 | 0.5 | 1.16 | 0.74, 1.82 | 0.5 |
| Critical care | 0.99 | 0.63, 1.57 | >0.9 | 0.98 | 0.62, 1.55 | >0.9 |
| Inpatient psychiatry | 0.66 | 0.25, 1.74 | 0.4 | 0.67 | 0.25, 1.75 | 0.4 |
| Others | 0.91 | 0.60, 1.37 | 0.6 | 0.86 | 0.57, 1.30 | 0.5 |
| Self-rated health |  |  |  |  |  |  |
| Excellent | — | — |  | — | — |  |
| Very good | 1.38 | 0.93, 2.04 | 0.11 | 1.35 | 0.91, 1.99 | 0.14 |
| Good or below | 1.54 | 0.96, 2.48 | 0.072 | 1.56 | 0.98, 2.50 | 0.063 |
| Any comorbidities | 1.15 | 0.78, 1.70 | 0.5 | 1.12 | 0.76, 1.66 | 0.6 |
| Household condition |  |  |  |  |  |  |
| Living alone | — | — |  | — | — |  |
| Living with others, no school-age children | 1.08 | 0.61, 1.89 | 0.8 | 1.07 | 0.61, 1.88 | 0.8 |
| Living with others, 1+ school-age children | 1.53 | 0.85, 2.77 | 0.2 | 1.48 | 0.82, 2.68 | 0.2 |
| Calendar month of taking survey |  |  |  |  |  |  |
| January | — | — |  | — | — |  |
| February | 1.52 | 0.96, 2.41 | 0.076 | 1.54 | 0.97, 2.43 | 0.068 |
| March | 0.51 | 0.29, 0.89 | 0.018 | 0.47 | 0.27, 0.83 | 0.009 |
| April | 0.12 | 0.06, 0.27 | <0.001 | 0.11 | 0.05, 0.25 | <0.001 |
| May | 0.48 | 0.27, 0.85 | 0.011 | 0.39 | 0.22, 0.70 | 0.002 |
| Months since last survey |  |  |  |  |  |  |
| 1 | — | — |  | — | — |  |
| 2 | 1.54 | 0.98, 2.43 | 0.063 | 1.59 | 1.01, 2.51 | 0.046 |
| 3+ | 4.20 | 2.48, 7.09 | <0.001 | 4.58 | 2.70, 7.74 | <0.001 |
| Months since recent COVID vaccine |  |  |  |  |  |  |
| 9+ months or never report | — | — |  | — | — |  |
| 3-9 months | 1.04 | 0.69, 1.56 | 0.9 | 1.13 | 0.75, 1.71 | 0.6 |
| 0-3 months | 0.82 | 0.45, 1.49 | 0.5 | 0.86 | 0.47, 1.57 | 0.6 |
| Months since recent SARS-CoV-2 infection |  |  |  |  |  |  |
| No COVID reported yet | — | — |  | — | — |  |
| 9+ months | 0.64 | 0.37, 1.10 | 0.10 | 0.60 | 0.35, 1.03 | 0.066 |
| 3-9 months | 0.07 | 0.01, 0.36 | 0.002 | 0.07 | 0.01, 0.36 | 0.001 |
| 0-3 months | 0.31 | 0.10, 0.97 | 0.045 | 0.29 | 0.09, 0.92 | 0.036 |
| Recent illness |  |  |  |  |  |  |
| No illness | — | — |  | — | — |  |
| Only 1 episode | 1.22 | 0.79, 1.89 | 0.4 | 1.26 | 0.81, 1.95 | 0.3 |
| 2+ episodes | 1.20 | 0.56, 2.56 | 0.6 | 1.30 | 0.61, 2.77 | 0.5 |

## Table S2. Principal component analysis (PCA) loadings for social activities

|  | PC1 | PC2 | PC3 |
| --- | --- | --- | --- |
| Socialized at a home indoors with non-household members | -0.2867 | 0.1919 | 0.5880 |
| Socialized outdoors with non-household members | -0.3881 | 0.4503 | 0.0474 |
| Socialized or attended an indoor event in public (i.e., concert/movies/sporting) | -0.4321 | -0.4497 | -0.1262 |
| Socialized or attended an outdoor event in public (i.e., concert/movies/sporting) | -0.4129 | 0.1017 | -0.3108 |
| Attended in-person religious services | -0.2079 | -0.6022 | 0.0201 |
| Went to a store (i.e., grocery, retail, etc.) | -0.2067 | -0.0654 | 0.0547 |
| Went to a gym or fitness center | -0.2735 | 0.0785 | 0.5277 |
| Ate indoors at a restaurant | -0.4323 | -0.1363 | -0.0909 |
| Traveled on an airplane | -0.2497 | 0.3954 | -0.4993 |

PC: principal component

# Survey Instrument

##
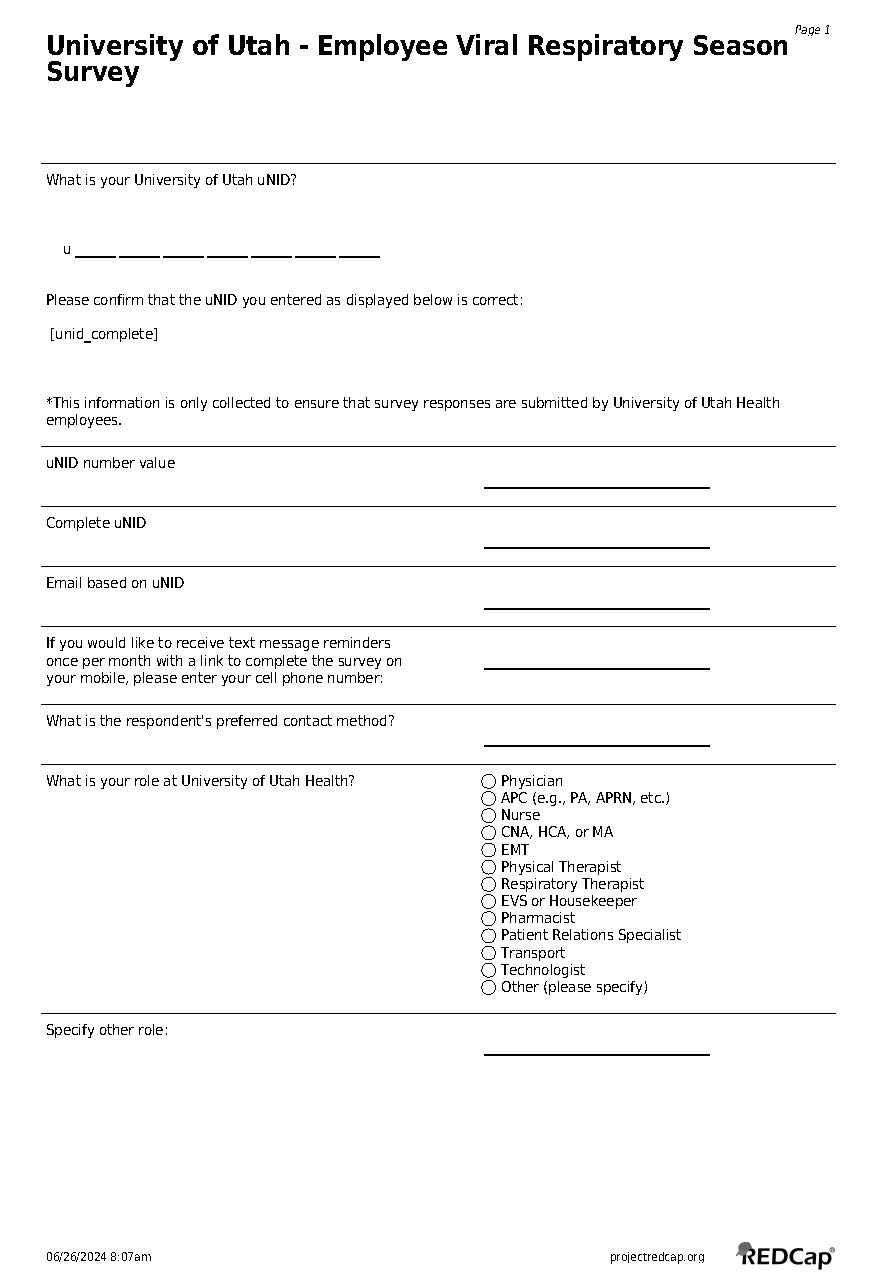
Baseline Survey

**
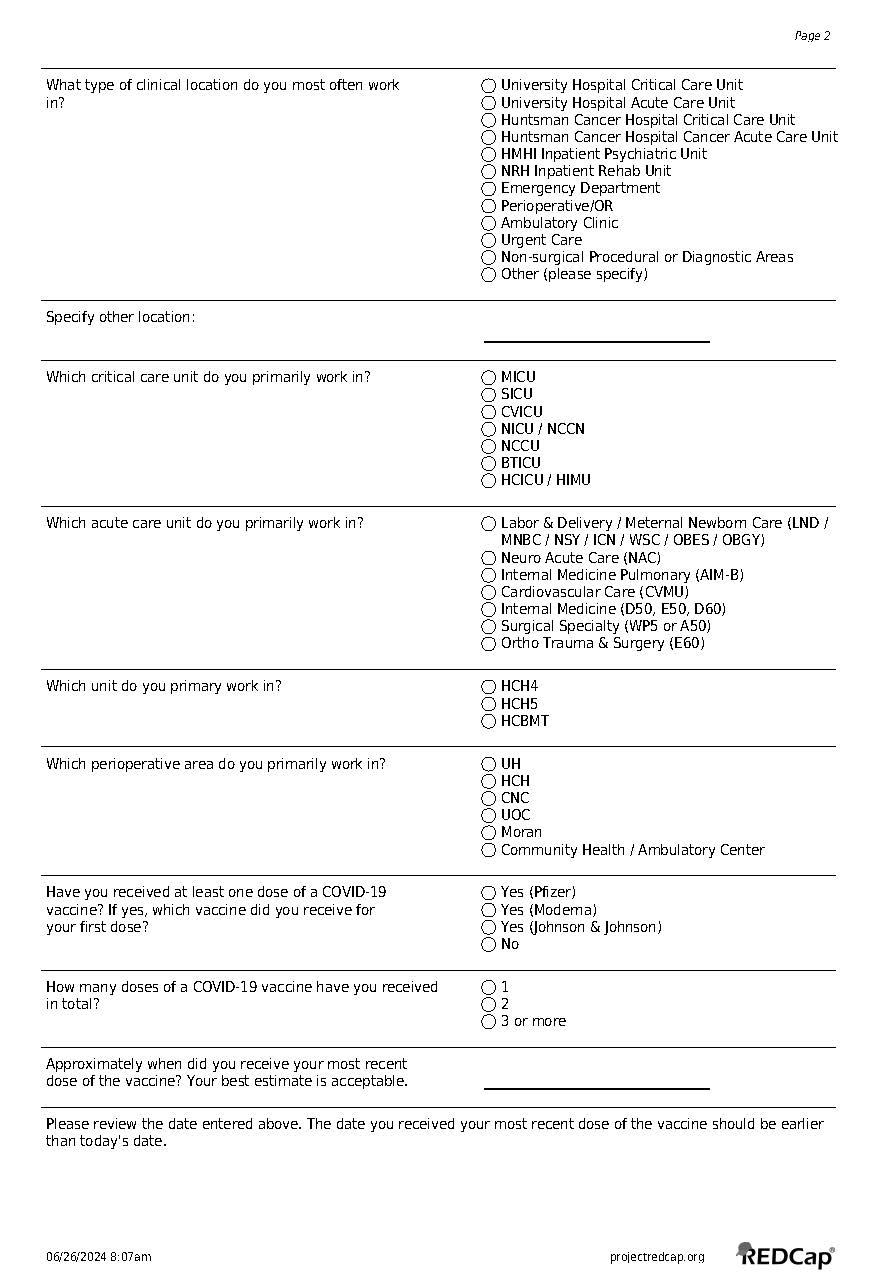

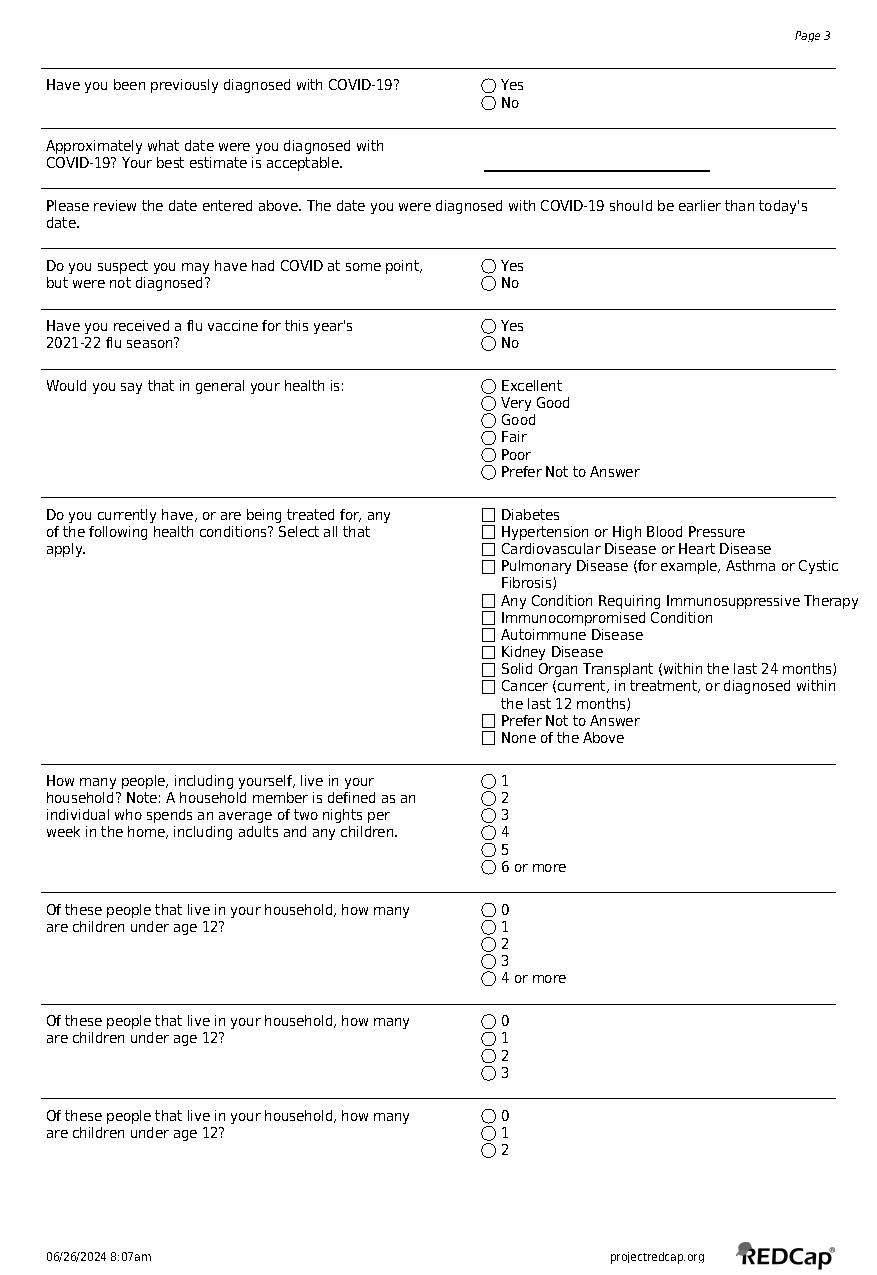

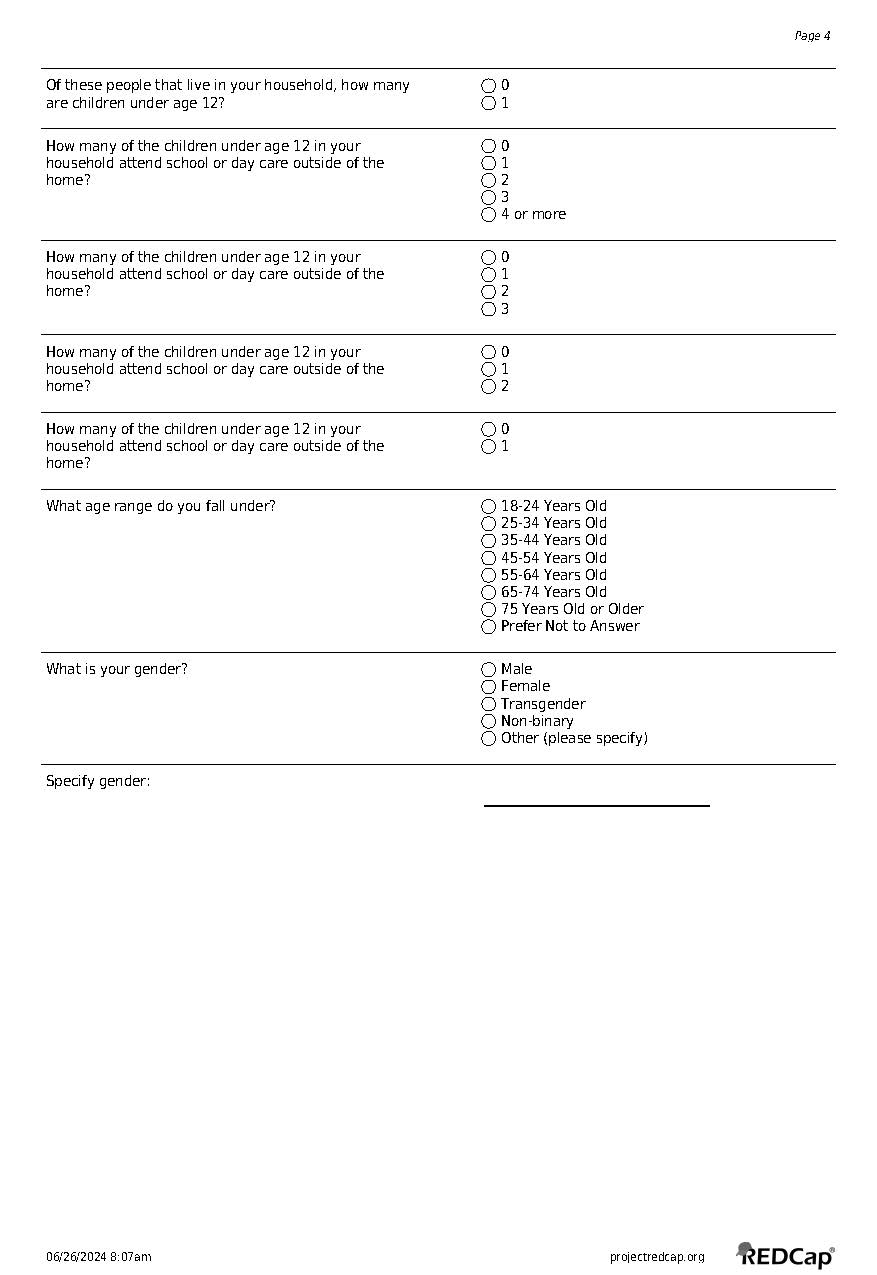
**

##
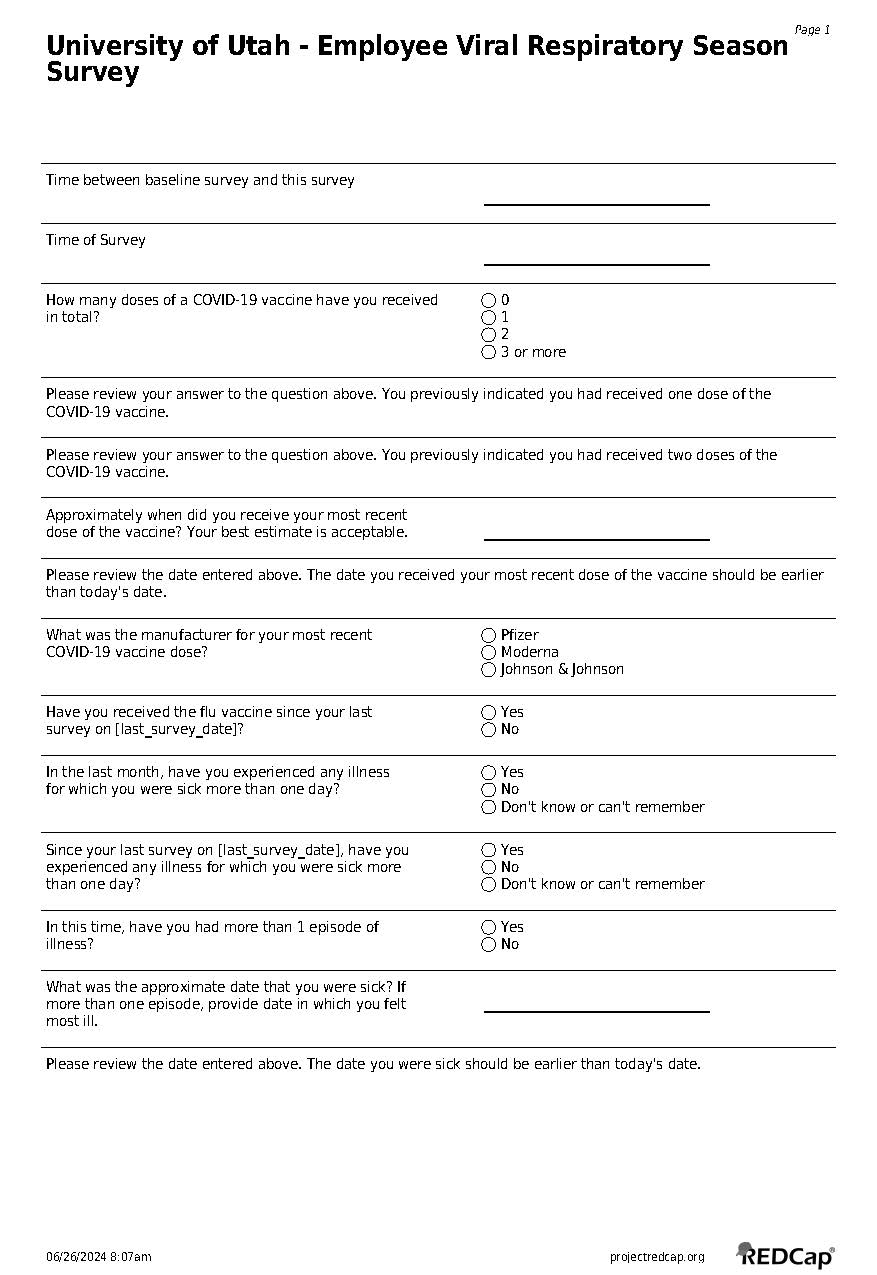
Monthly Survey


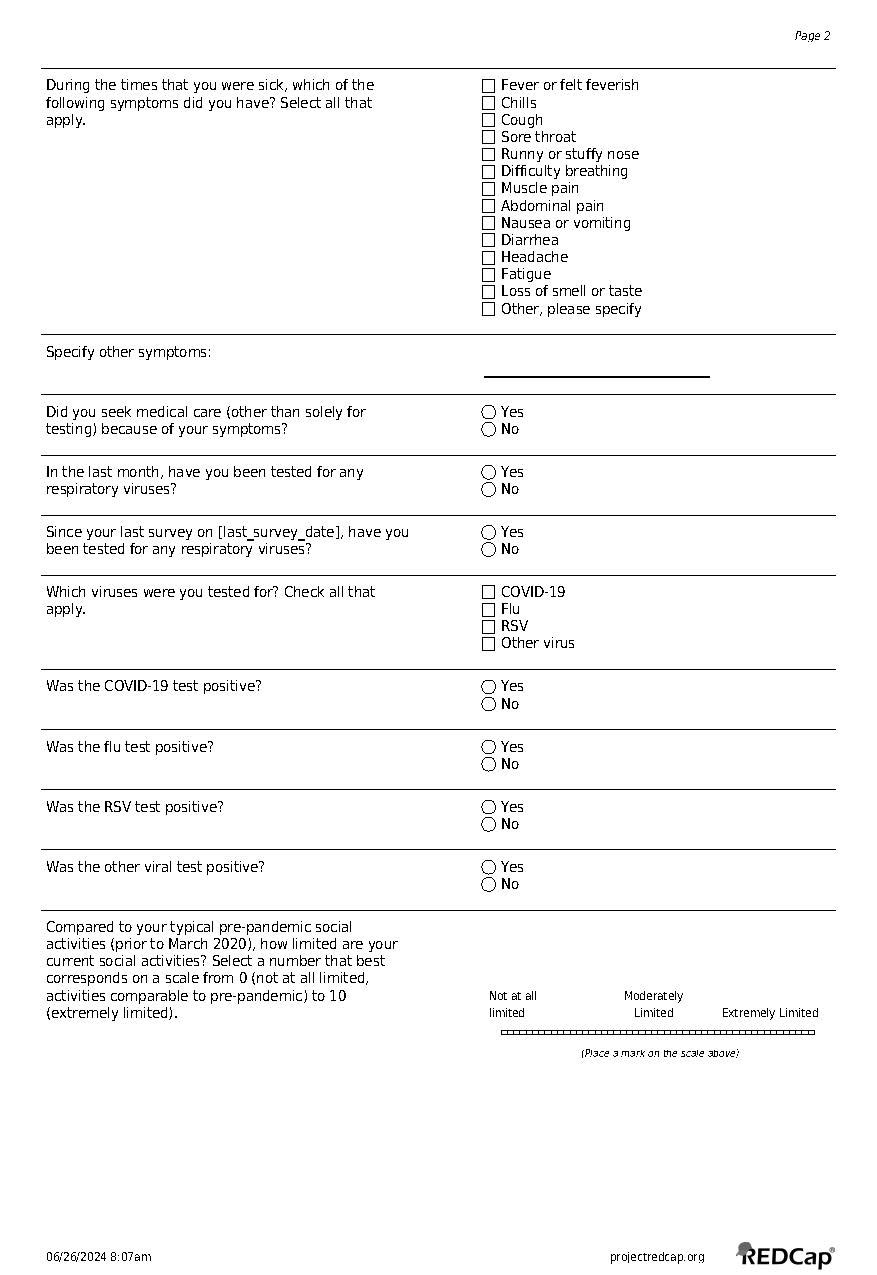

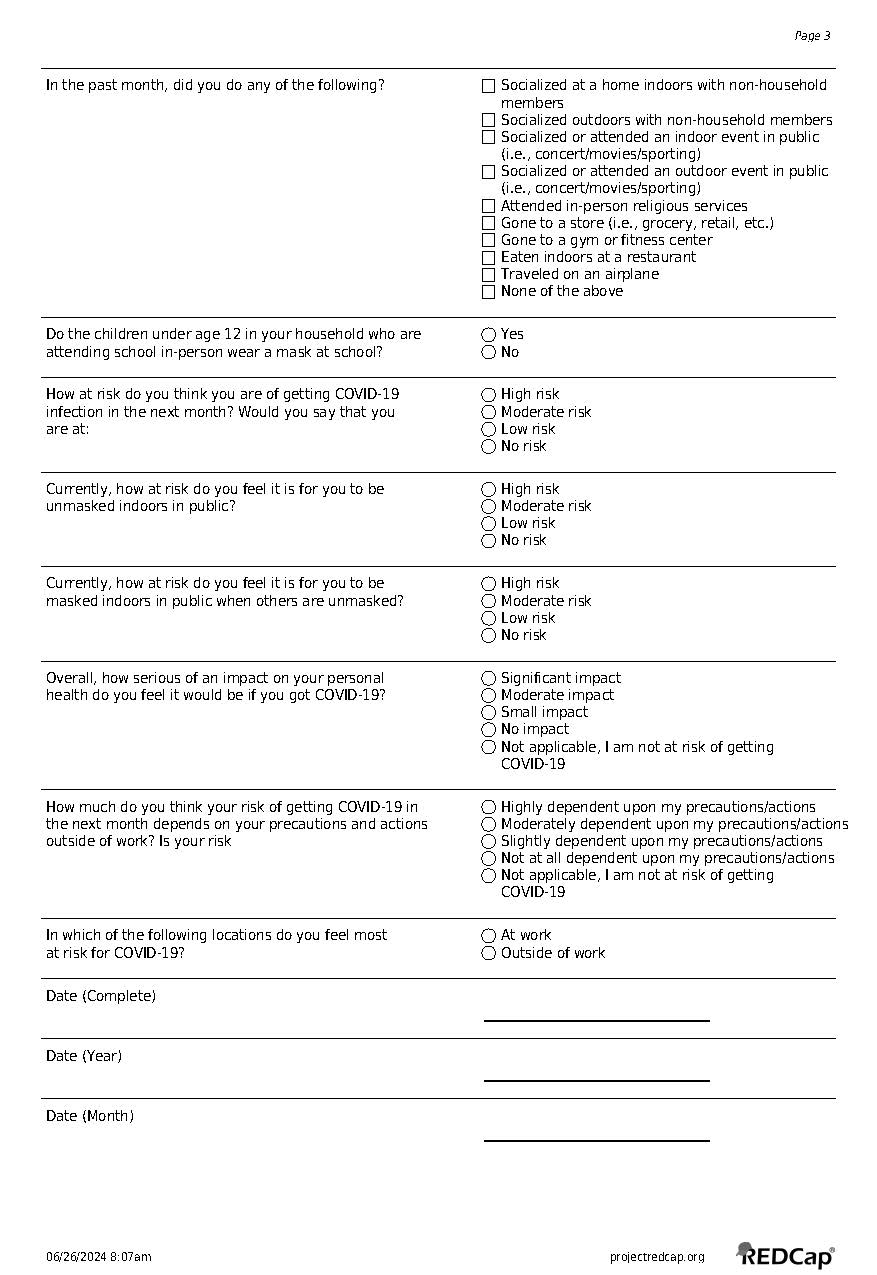

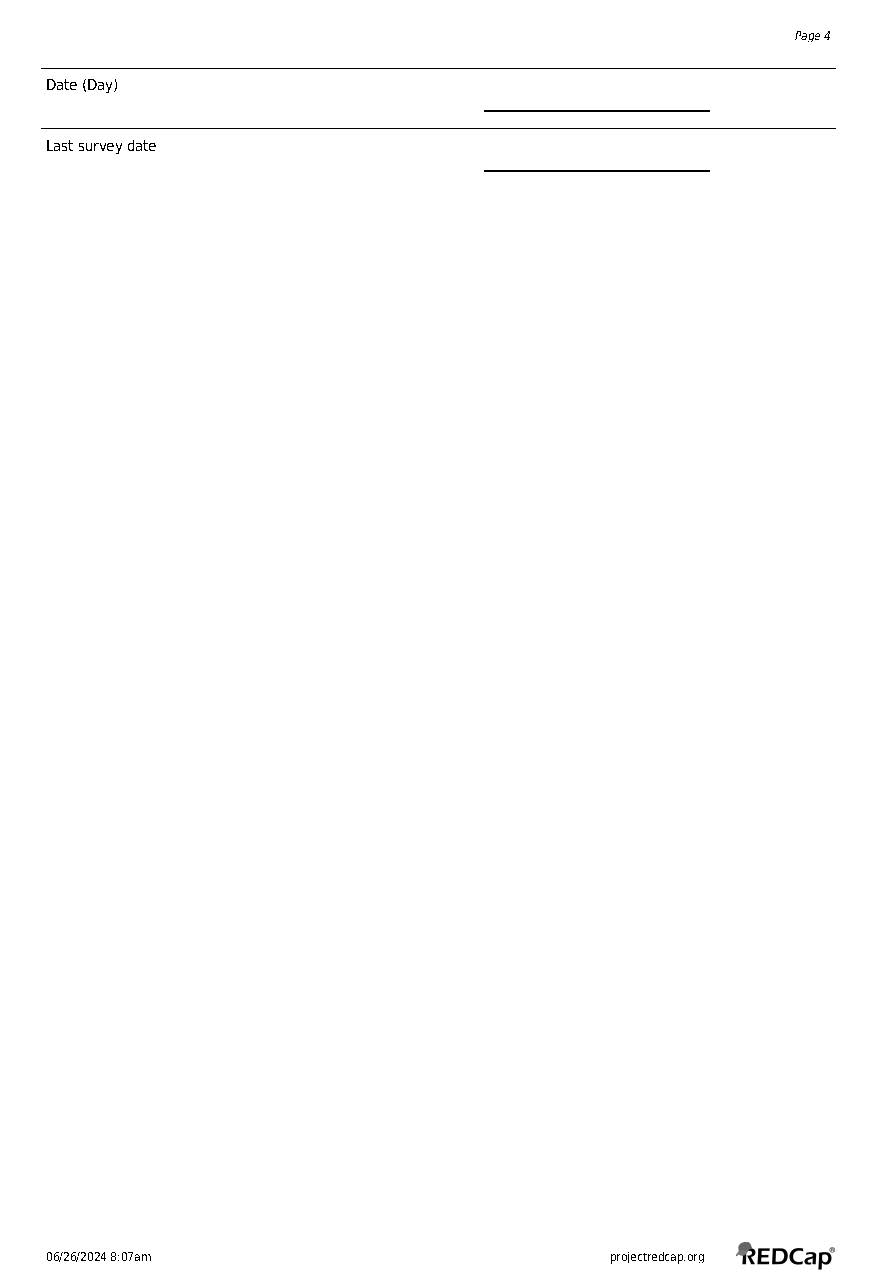

Supplement: Shoemaker et al. supplementary material [file S2732494X24004856sup001.docx]
